# An efficient hybrid reliability analysis method with application to the harmonic drive

**DOI:** 10.1371/journal.pone.0330569

**Published:** 2025-08-21

**Authors:** Jingqi Cui, Di Zuo, Xiaoxi Men, Chongshuai Wang

**Affiliations:** 1 School of Mechanical Engineering, Dalian Jiaotong University, Dalian, China; 2 School of Transportation Engineering, Dalian Jiaotong University, Dalian, China; 3 State Key Laboratory of Intelligent Power Distribution Equipment and System, School of Electrical Engineering, Hebei University of Technology, Tianjin, China; Aalto University, FINLAND

## Abstract

The harmonic drive is a new type of high-efficiency gear transmission system, widely used in precision machinery, robotics, and aerospace due to its compact structure and high transmission accuracy, and the multiple uncertain parameters in it will seriously affect its transmission performance. In this paper, the limit state functions for multiple failure modes of harmonic drive are established based on the stress-strength interference theory. Considering the hybrid uncertainties in the harmonic drive, the modified chaos control method and multiplicative dimension reduction method are coupled to solve the probabilistic and interval reliabilities continuously. The proposed model is simple and easy to implement, and the failure probabilities of different failure modes of harmonic reducers can be accurately and quickly evaluated. Furthermore, the differences in the influence of strength on different failure modes are demonstrated by the results of hybrid uncertainty analysis.

## 1 Introduction

The harmonic drive is a kind of gear transmission system and its unique transmission principle endows the harmonic drive with the advantages of small size, large transmission ratio, and strong load bearing capacity [1,2]. Currently, it has been widely used in industries such as industrial robots, aerospace, and medical treatment [3,4]. During the operation process, uncertain parameters in the structure will significantly affect the normal operation of the harmonic drive and even lead to its failure [5,6]. Therefore, studying the reliability analysis of the harmonic drive is of great significance.

In uncertainty analysis, uncertainty can generally be divided into three types, namely probabilistic uncertainty, interval uncertainty, and fuzzy uncertainty [7–9]. Much research has already been carried out on single uncertainty problems. However, when there are multiple uncertain variables in one structure, the response of the structure will become more complicated [10]. At present, some scholars have studied the hybrid uncertainty problems. For instance, Eldred [11] combined the stochastic expansion with the optimization-based interval estimation method and proposed a new hybrid uncertainty analysis algorithm. Lu *et al*. [12] transformed variables based on entropy equality information and studied the probabilistic-interval reliability analysis. Wu *et al*. [13] combined sampling with interval analysis and proposed the unified interval stochastic sampling method to study the stability assessment problem of engineering structures under hybrid uncertainties. Moreover, Shah *et al*. [14] constructed a surrogate model by using the non-intrusive polynomial chaos technique and proposed an efficient robust optimization algorithm for wing design under hybrid uncertainties. Yao *et al*. [15] decomposed the complex nested problem into deterministic optimization and uncertainty analysis sub-problems in the research on the reliability optimization problem under hybrid uncertainties and proposed the sequential optimization and hybrid uncertainty analysis method based on probability and evidence theory. Some other scholars have also explored the hybrid uncertainty problem involving probability and fuzziness. Adduri and Penmetsa [16,17] proposed a method leveraging the response surface model and the possibility function transformation technique. This approach effectively reduced the computational burden when handling the hybrid uncertainty of probability and fuzziness. Song *et al*. [18] put forward a novel uncertainty importance measure index, aiming at the issue that structural reliability and response are influenced by random and fuzzy variables. Yang *et al*. [19] devised a hybrid genetic algorithm to explore the random and fuzzy uncertainties present in railway freight planning. Tang [20] proposed a data-driven probabilistic fuzzy system modeling method. Liu [21] proposed a unified quantification framework for hybrid uncertainties grounded in the imprecise probability theory and established a reliability model accounting for hybrid uncertainties and failure correlations. Furthermore, Meng [22] further investigated the hybrid uncertainty problem that encompasses randomness, fuzziness, and intervals.

In terms of the field of uncertainty analysis in harmonic drive, Leon *et al*. [23] conducted a statistical analysis of the influence of tooth geometry in the performance of a harmonic drive, and Hrcek *et al*. [24] investigated the global sensitivity analysis of chosen harmonic drive parameters affecting its lost motion. The reliability analysis of harmonic drive is also investigated, such as, Zhang *et al*. [25] calculated the reliability of the filtering reducer under different temperatures and impact loads; Sun *et al*. [26] proposed a fuzzy reliability analysis method for harmonic drive based on the failure intensity model of harmonic drive; Li *et al*. [27] proposed a modeling method for the remaining fatigue life and a reliability analysis method for harmonic drive considering multisource uncertainties based on the wear allowance model of the flexible gear tooth surface. Considering the dynamic reliability, Zhang *et al*. [28] studied the dynamic reliability of harmonic speed limiters using the polynomial chaos expansion method; Zhao *et al*. [29] took into account the harmonic hysteresis phenomenon generated by the harmonic drive and proposed a new method for analyzing the dynamic reliability of the transmission space and the parameter sensitivity of the harmonic drive.

In the above study, the reliability analysis of harmonic drive only considered the influence of a single uncertainty parameter. However, in practical engineering, due to cognitive limitations and random uncertainties, structural components may simultaneously contain both probabilistic and interval uncertainty parameters. This significantly affects the reliability prediction results of harmonic reducers, leading to inaccuracies in structural life prediction. In this paper, a fast hybrid uncertainty analysis method is employed to deal with the probability and interval variables. The hybrid reliability of probability and interval is decomposed into iterative solutions for probabilistic reliability and interval uncertainty. Specifically, the modified chaos control method (MCCM) and the multiplicative dimensionality reduction method (MDRM) are adopted to handle probabilistic and interval uncertainty analyses respectively. Then, based on the proposed method, the hybrid uncertainty analysis of the three failure modes of the harmonic drive is conducted.

The remainder of this paper is organized as follows: Sect 2 presents the basic theory of the proposed model. Sect 3 introduces three failure modes of harmonic drive, Sect 4 shows the numerical examples, and discussions and conclusions are provided in Sect 5.

## 2 Model and method

### 2.1 Hybrid reliability analysis method based on MCCM-MDRM method

#### 2.1.1 Basic definition.

Considering a system with random and interval variables, let $X=[X_{1},X_{2},...,X_{n}]$ stands for an independent *n*-dimensional probabilistic uncertain input vector, $Y=[Y_{1}^{I},Y_{2}^{I},...,Y_{m}^{I}{]}^{⊤}$ denote an independent *m*-dimensional interval uncertain vector, and z(X,Y) represent the output vector. Then, a failure criterion can be established as

$$z(X,Y)>z^{\max}$$ (1)

where the $z^{\max}$ represent a maximally admissible value.

Based on the definition provided in Eq (1), a limit state function can be formulated as:

$$\text{g}(X,Y)=z^{\max}-z(X,Y)$$ (2)

Then, the Probability of Failure (PoF) can be defined as

$$P_{f}=\mathbb{P}(\text{g}(X,Y)<0)$$ (3)

in which ℙ(·) is the probability of (·).

### 2.2 Hybrid reliability analysis.

When solving structural failure problems, the solution of PoF is usually transformed into the solution of reliability index *β* to avoid the huge computational time of Monte Carlo simulation (MCS). The geometric significance of the reliability index *β* is the minimum distance from the origin of the standard normal space to the limit state surface, and the relationship between PoF *P*_*f*_ and the reliability index *β* can be expressed as [30]:

$$P_{f}=\Phi (-\beta )$$ (4)

where the Φ(·) is the cumulative distribution function of the standard normal distribution.

**Fig 1:**
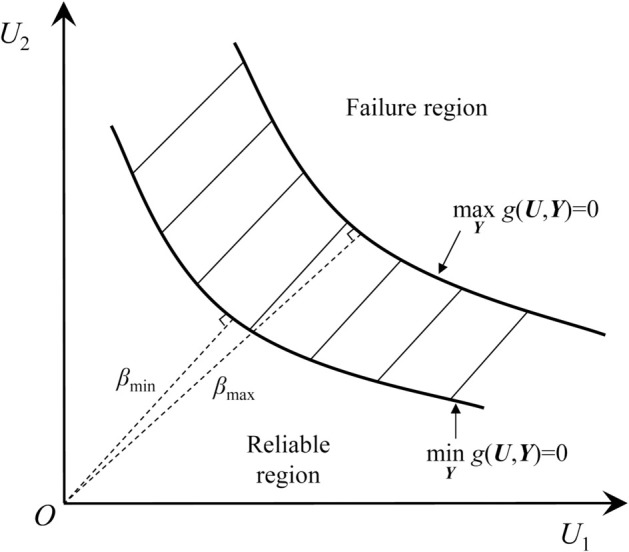
Diagram of the limit state band within a two-dimensional standard state space.

Due to the simultaneous existence of random and interval variables, the reliability index *β* of the structure transforms from a deterministic value to an interval $\left[\beta_{\min},\beta_{\max}\right]$, as shown in Fig 1.

In practical engineering, the maximum PoF $P_{f}^{\max}$ is more concerned. Thus, only the minimum reliability index $\beta_{\min}$ is considered in this paper, which can be solved by the following optimization problem:

$$\left\{ \begin{array}{c} \beta_{\min}=\underset{U}{\min}\left\|U\right\|\\ s.t.\underset{Y}{\min}\text{ g}(U,Y)=0 \end{array}\right.$$ (5)

in which U is the probability variable in standard normal distribution space, which can be obtained through Rosenblatt transformation [31] as

$$\left\{ \begin{array}{c} \phi \left(U\right)=F_{X}\left(X,Y\right)\\ X=F_{X}^{-1}(\phi \left(U\right),Y) \end{array}\right.$$ (6)

In solving the problem definition in Eq (6), iterative solution of probabilistic reliability and interval uncertainty is involved. In this paper, a chaos control method is adopted to deal with the probabilistic reliability analysis, and the multiplicative dimensional reduction method is employed in interval analysis.

#### 2.2.1 Modified chaos control method.

Assuming the random vector obtained during the *k*-th iteration is $U^{k}$ and the interval vector is $Y^{k}$. In probabilistic reliability analysis, the interval vector $Y^{k}$ remains fixed in the iteration. Moreover, based on the HL-RF (Hasofer-Lind and Rackwitz-Fiessler) method [32], the random vector in the (k+1)-th iteration can be computed as:

$$\left\{ \begin{array}{c} U^{k+1}=-\beta^{k+1}\frac{\nabla \text{g}(U^{k},Y^{k})}{\left||\text{g}(U^{k},Y^{k})|\right|}\\ \beta^{k+1}=\frac{\text{g}(U^{k},Y^{k})-\nabla \text{g}(U^{k},Y^{k}{)}^{⊤}U^{k}}{\left||\text{ g}(U^{k},Y^{k})|\right|} \end{array}\right.$$ (7)

When the nonlinearity degree of the limit state function is high, the phenomenon of oscillation will occur when the HL-RF method is adopted, resulting in the failure of convergence of the results. In line with the work of Yang [33], the CCM is introduced to govern the convergence of the HL-RF iterative algorithm. Subsequently, Eq (7) can be rewritten as:

$$\left\{ \begin{array}{c} U^{k+1}=U^{k}+\lambda C(h(U^{k},Y^{k})-U^{k})\\ h(U^{k},Y^{k})=-\beta^{k+1}\frac{\nabla \text{g}(U^{k},Y^{k})}{\left||\nabla \text{g}(U^{k},Y^{k})|\right|} \end{array}\right.$$ (8)

In Eq (8), λ∈(0,1) is the control factor. C is involuntary matrix, and is unit tensor in this paper. For more detail descriptions, the readers can refer to the work of Pingel *et al*. [34].

In CCM, the step size remains constant, which leads to low calculation efficiency. To address this issue, a modified chaos control method (MCCM) is adopted [35], and the new iteration can be expressed as:

$$\left\{ \begin{array}{c} U^{k+1}=\beta^{k+1}\frac{n(U^{k+1})}{\left||n(U^{k+1})|\right|}\\ n(U^{k+1})=U^{k}+\lambda C(h(U^{k},Y^{k})-U^{k})\\ h(U^{k},Y^{k})=-\beta^{k+1}\frac{\nabla \text{g}(U^{k},Y^{k})}{\left||\nabla \text{g}(U^{k},Y^{k})|\right|} \end{array}\right.$$ (9)

in which the $n^{k}$ is the unit direction vector in the direction of the negative derivative of the function $\text{g}(U^{k},Y^{k})$. In MCCM, the iterative process has been relaxed, thus significantly improving the computational efficiency.

#### 2.2.2 Multiplicative dimensional reduction method.

Based on the Multivariate Dimension Reduction Method (MDRM), the limit state function $\text{g}(U^{k\;+\;1},Y)$ within the (k+1)-th iteration can be expanded at the midpoint of the interval and presented in the form of a product of single-variable functions, as shown below:

$$\text{g}(U^{k+1},Y)\approx \text{g}(U^{k+1},Y^{c}{)}^{1-m}\prod_{j=1}^{m}{\text{g}}_{j}(U^{k+1},Y_{-j}^{c})$$ (10)

where $Y^{c}=[Y_{1}^{c},Y_{2}^{c},...,Y_{m}^{c}]$ is the midpoint of the interval, and $Y_{-j}^{c}=[Y_{1}^{c},...,Y_{j-1}^{c},Y_{j},Y_{j+1}^{c},...,Y_{m}^{c}]$ is the vector except the *Y*_*j*_.

Through the second-order Taylor expansion at the midpoint of its interval, ${\text{g}}_{j}(U^{k+1},Y_{-j}^{c})$ can be expressed as

$${\text{g}}_{j}(U^{k+1},Y_{-j}^{c})=\text{g}(U^{k+1},Y^{c})+\frac{\partial {\text{g}}_{j}(U^{k+1},Y^{c})}{\partial Y_{j}}\delta_{j}Y_{j}^{r}+\frac{\partial^{2}{\text{g}}_{j}(U^{k+1},Y^{c})}{\partial Y_{j}^{2}}\frac{(\delta_{j}Y_{j}^{r}{)}^{2}}{2}$$ (11)

where $Y_{j}^{r}$ is the radius of the interval variable, and $\delta_{j}=[-1,1]$.

Focusing on Eq (11), and it can be rewritten in the form of a quadratic function as:

$${\text{g}}_{j}(U^{k+1},Y_{-j}^{c})=\Psi_{j}(\delta_{j})=a_{j}\delta_{j}^{2}+b_{j}\delta_{j}+c_{j}$$ (12)

in which

$$\left\{ \begin{array}{c} a_{j}=\frac{\partial^{2}{\text{g}}_{j}(U^{k+1},Y^{c})}{\partial Y_{j}^{2}}\\ b_{j}=\frac{\partial {\text{g}}_{j}(U^{k+1},Y^{c})}{\partial Y_{j}}Y_{j}^{r}\\ c_{j}=\text{g}(U^{k+1},Y^{c}) \end{array}\right.$$ (13)

As demonstrated in Eq (12), the minimum value of the limit state function $\text{g}(U^{k+1},Y)$ within the interval $Y\in \left[Y^{L},Y^{U}\right]$ and the corresponding value of $\delta_{j}$ can be obtained. As a result, the interval vector for the (k+1)-th iteration can be expressed as

$$Y^{k+1}=Y^{c}+\delta Y^{r}$$ (14)

When the convergence criteria in Eq (15) are satisfied, the iteration is stopped, and at this point $U^{k+1}$ represents the most probable point for the minimum reliability index $\beta_{\min}=||U^{k+1}||$.

$$\left\{ \begin{array}{c} \frac{\left||U^{k+1}-U^{k}|\right|}{\left||U^{k}|\right|}\leq \epsilon_{1}\\ \text{g}(U^{k+1},Y^{k+1})\leq \epsilon_{2} \end{array}\right.$$ (15)

where $\epsilon_{1}$ and $\epsilon_{2}$ are the predefined tolerance. In this paper, the tolerances are set as $\epsilon_{1}=\epsilon_{2}=10^{-6}$.

## 3 Fatigue strength of flexspline

**Fig 2:**
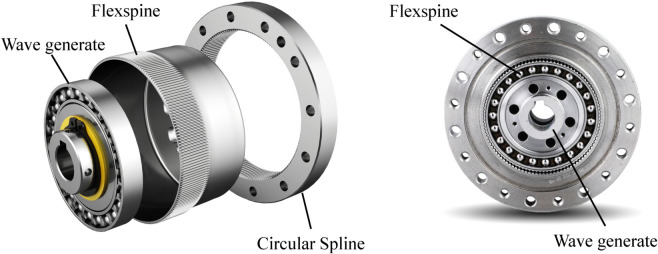
Schematic diagram of harmonic drive.

The harmonic drive mainly includes a wave generator, flexspline, and the circular spline, and its structure diagram is shown in Fig 2. The main failure modes of the harmonic drive involve fatigue fracture of the flexspline, contact fatigue of the flexspline, and tooth surface wear fatigue of the flexspline [36]. In this paper, these three modes are analyzed.

### 3.1 Fatigue fracture of flexspline

According to the work of Li [37], the bidirectional stable variable stress state is adopted to assess the fatigue fracture strength of the flexspline as:

$$S=\frac{S_{\sigma }S_{\tau }}{\sqrt{S_{\sigma }^{2}+\gamma_{z}S_{\tau }^{2}}}$$ (16)

where

$$S_{\sigma }=\frac{\sigma_{-1}}{\sigma_{a}}$$ (17)

$$S_{\tau }=\frac{\tau_{-1}}{\tau_{a}+0.2\tau_{m}}$$ (18)

in which the $\sigma_{-1}$ and $\tau_{-1}$ are the bending fatigue limit and shear fatigue limit under symmetric cyclic loading, respectively. $\sigma_{a}$ and $\tau_{a}$ are the stress amplitudes of the normal and shear stresses, respectively. $\tau_{m}$ is the average stresses of the shear stresses, and $\gamma_{z}=0.7$ is the influence coefficient of axial normal stress on the fatigue fracture failure of the flexible gear [39].

Following the work of Zuo *et al*. [39], the detail expression for Eqs (17), (18) are given as

$$\left\{ \begin{array}{c} S_{\sigma }=\frac{r_{m}^{2}\sigma_{-1}}{K_{\sigma }K_{M}K_{d}C_{\sigma }w_{0}E\delta_{t}}\\ S_{\tau }=\frac{4\pi r_{m}^{2}\delta_{t}L\tau_{-1}}{K_{\tau }K_{d}\left(K_{u}TL+2\pi r_{m}\delta_{t}^{2}K_{M}C_{\tau }w_{0}E\right)} \end{array}\right.$$ (19)

In Eq (19), $K_{\sigma }$ represents the normal stress concentration coefficient, while $K_{\tau }$ denotes its shear stress counterpart. The symbol *K*_*M*_ refers to the stress growth coefficient induced by the flexspline’s shape distortion, whereas *K*_*u*_ corresponds to the shear stress distribution unevenness coefficient. Additionally, *K*_*d*_ stands for the dynamic loading coefficient. Here, *w*_0_ signifies the maximum radial displacement of the flexspline. The terms $C_{\sigma }$ and $C_{\tau }$ indicate the normal and shear stress coefficients, respectively. Moreover, $\delta_{t}$ represents the flexspline wall thickness, *E* denotes the elastic modulus, *T* is the input torque, *r*_*m*_ is the radius of the mid-surface of the flexspline, and *L* refers to the flexspline’s length. For a more detailed description, readers can refer to our previous work [39].

In the design of harmonic drives, a safety factor *S*_*f*_ is introduced to guarantee reliability and safety. The limit state function that depicts the fatigue fracture of the flexspline can be expressed as follows:

$$g(X,Y)=\frac{S_{\sigma }S_{\tau }}{\sqrt{S_{\sigma }^{2}+\gamma_{z}S_{\tau }^{2}}}-S_{f}$$ (20)

### 3.2 Contact fatigue of the flexspline

When designing the harmonic drive, the contact fatigue life of the flexspline is computed using the general bearing fatigue life theory, and it can be presented as [38–40]:

$$L_{h}=\frac{10^{6}}{60n_{r}}{\left(\frac{C_{d}}{P}\right)}^{\eta }$$ (21)

where *L*_*h*_ represents the fatigue life of the flexspline. The relative rotational speed between the inner and outer races of the elliptical bearing is denoted by *n*_*r*_. The rated dynamic load capacity of the flexspline is *C*_*d*_. The fatigue life exponent is represented by *η*, and for ball bearings, η=3. The equivalent load on the flexspline is *P*, and it can be expressed as follows:

$$P=VF_{r}f_{p}f_{t}$$ (22)

where *V* stands for the seat rotation coefficient, and typically V=1.2. The load coefficient and temperature coefficient are denoted by *f*_*p*_ and *f*_*t*_ respectively. The radial load on the flexspline is *F*_*r*_, and it can be expressed as follows:

$$F_{r}\approx 1.08K_{r}\frac{2T}{Ud_{1}\text{cos}\alpha }$$ (23)

where *K*_*r*_ is the transmission coefficient, *U* represents the wave number, *d*_1_ is the diameter of the flexspline’s pitch circle, and *α* denotes the tooth angle of the involute gear. For the cam-type wave generator harmonic drive, the value of the transmission coefficient is set as *K*_*r*_ = 0.35 [39]. When $\alpha =20^{\circ }$ and *U* = 2, by substituting Eqs (23), (22) into Eqs (21), (21) can be rewritten as:

$$L_{h}=\frac{10^{6}}{60n_{r}}{\left(\frac{C_{d}d_{1}}{1.15VK_{r}f_{p}f_{t}T}\right)}^{3}$$ (24)

Based on the flexspline dimensions of the harmonic drive, the rated dynamic load can be calculated as [38,39]:

$$C_{d}=f_{c}{\left(i_{s}\text{cos}\alpha_{\text{0}}\right)}^{0.7}z_{b}^{2/3}d_{g}^{1.8}$$ (25)

where *f*_*c*_ represents the geometry of the flexspline. The number of ball columns is denoted by *i*_*s*_, and in this case *i*_*s*_ = 1. The initial contact angle between the ball and the inner and outer rings of the bearing is $\alpha_{0}=12^{\circ }$. The number of balls is *z*_*b*_ = 23, and the diameter of the ball is given by $d_{g}=0.09d_{1}$.

By substituting Eq (25) into Eq (24), and assuming that the contact fatigue limit of the flexspline corresponds to the life of the harmonic drive of $L_{h}^{e}$ hours, the function for the contact fatigue failure mode of the flexspline can be expressed as follows:

$$g_{2}(𝐗)=\frac{5.62}{n_{r}}{\left(\frac{d_{1}^{2.8}}{T}\right)}^{3}\times 10^{-3}-L_{h}^{e}$$ (26)

### 3.3 Tooth surface wear fatigue of the flexspline

The calculation of the tooth surface wear fatigue of the flexspline is based on the specific pressure *p* [39]. As proposed by Shen [41], the expression for the specific pressure *p* of the tooth surface under operating conditions is as follows:

$$p=\frac{2000TK}{d_{1}h_{n}bz_{v}}$$ (27)

where *K* denotes the load factor; *h*_*n*_ represents the maximum engagement depth; *b* is the tooth width; and $z_{v}$, the equivalent number of teeth along the working section of the full mesh tooth contour, can be determined by the following expression:

$$z_{v}=\frac{\epsilon z_{1}}{4}$$ (28)

By substituting Eq (28) into Eq (27) and taking into account the allowable specific pressure *p* of the flexspline material, the function representing the failure mode of the tooth surface wear fatigue of the flexspline can be written as:

$$g_{3}(𝐗)=\frac{8000TK}{d_{1}h_{n}b\epsilon z_{1}}-p$$ (29)

## 4 Numerical examples

In this section, different examples are tested to show the performance of the proposed method and the hybird uncertainty analysis results of the harmonic drive. In MCS 10^5^ is used for the probability variable sample. In Tables 2, 8 and 12, for the normal distribution, the parameters 1 and 2 denote the mean and standard deviation, respectively; For the interval variable, the parameters 1 and 2 denote the lower and upper bounds, respectively.

### 4.1 Verification case

#### 4.1.1 Case 1.

In this example, a simple example is tested to show the performance of the MCCM, which the limit state function is defined as [42]:

$$g(X)=x_{1}^{4}+2x_{2}^{4}-20$$ (30)

in which *x*_1_ and *x*_2_ are two uncertain variables obeying the normal distribution $\text{N}(10,5^{2})$.

**Fig 3:**
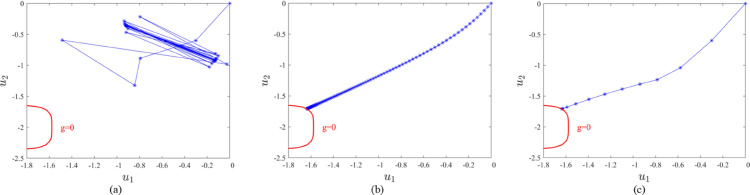
Iterative histories of different methods. (a) HL-RF method, (b) CCM method, (c) MCCM method.

Fig 3 shows the iterative histories of different methods. As illustrated in Fig 3, the HL-RF method exhibits oscillatory behavior and fails to converge to the design point. The method proposed in this paper MCCM-MDRM not only effectively suppresses the oscillations of random variables but also increases the iteration step size, thereby significantly improving the convergence speed.

#### 4.1.2 Case 2.

In this example, a more complex case is tested to demonstrate the performance of MCCM in handling multi-variable problems and non-normal distributions. The limit state function is defined as follows [42]:

$$g(X)=2+0.0015\sum_{i=1}^{9}x_{i}^{2}-3x_{10}$$ (31)

where $x_{1}\sim x_{3}$ follow the standard normal distribution *N*(0,1); $x_{4}\sim x_{6}$ follow the lognormal distribution LogNormal(0,1); $x_{7}\sim x_{9}$ follow the Gumbel distribution Gumbel(0,1). To evaluate the accuracy of the proposed method, a relative error *R*_*e*_ is defined as:

$$R_{e}=\left|\frac{P_{f}^{\text{PRE}}-P_{f}^{\text{MCS}}}{P_{f}^{\text{MCS}}}\right|\times 100\%$$ (32)

where $P_{f}^{\text{PRE}}$ denotes the PoF *P*_*f*_ predicted by different methods, and $P_{f}^{\text{MCS}}$ is the result obtained from MCS.

Table 1 presents the comparison results of different methods. It can be observed that both CCM and MCCM yield *P*_*f*_ with small errors, confirming the performance of the proposed method in addressing multi-variable problems and non-normal distributions.

**Table 1:** Comparison of the PoF *P*_*f*_ for different methods.

	MCS	CCM	MCCM
*P*_*f*_	0.2478	0.2520	0.2519
*R*_*e*_	-	1.69%	1.65%

### 4.2 Fatigue fracture of flexspline

In this example, the output torque *T*, bending fatigue limit $\sigma_{-1}$, and shear fatigue limit $\tau_{-1}$ are treated as interval variables, and other parameters are treated as random variables. The detailed distributions of these parameters are outlined in Table 2.

**Table 2:** The distribution of the uncertain variables used in assessing the fatigue fracture of flexspline of the harmonic drive.

Variable	Distribution parameter 1	Distribution parameter 2	Variable type
*L* (mm)	20	2	Normal
$\delta_{t}$ (mm)	0.7	0.007	Normal
*r*_*m*_ (mm)	25	0.25	Normal
*w*_0_ (mm)	0.25	0.0025	Normal
*γ*	0.7	0.007	Normal
$K_{\sigma }$	2.2	0.022	Normal
$K_{\tau }$	1.5	0.015	Normal
*K*_*M*_	1.4	0.014	Normal
*K*_*u*_	1.5	0.015	Normal
*K*_*d*_	1.1	0.011	Normal
$C_{\sigma }$	1.6	0.016	Normal
$C_{\tau }$	0.6	0.006	Normal
*E* (GPa)	210	2.1	Normal
*T* (N·m)	27	33	Interval
$\sigma_{-1}$ (MPa)	515	545	Interval
$\tau_{-1}$ (MPa)	285	315	Interval

Table 3 presents the results of MCS for different discretization numbers of interval variables, and the results demonstrated that the discretization number *N*_*t*_ = 20 is sufficient to guarantee the accuracy of MCS. Table 4 shows the results of AK-MCS under different convergence criteria. Strengthening the convergence criteria would significantly compromise the computational efficiency of AK-MCS, although accuracy would also improve. To balance the computational efficiency and accuracy of AK-MCS, its convergence condition was set to 10^−3^ in following comparisons.

**Table 3:** Comparison of the maximum probability of failure $P_{f}^{\max}$ from MCS under different discretization numbers of interval variables.

	*N*_*t*_ = 10	*N*_*t*_ = 20	*N*_*t*_ = 100
MCS	0.0202	0.0200	0.0199

**Table 4:** Comparison of AK-MCS results for different convergence criterion.

	10^−1^	10^−3^	10^−5^
Iteration times	28	60	238
$P_{f}^{\max}$	0.1032	0.1011	0.1010

**Fig 4:**
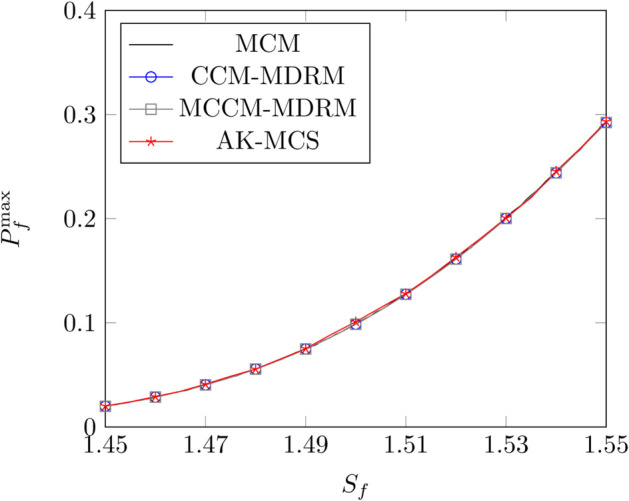
The variation of the maximum PoF $P_{f}^{\max}$ with the safety factor *S*_*f*_ for different methods.

Fig 4 show the results for different methods under the control parameter λ=0.1. The results in Fig 4 demonstrate that as the safety factor *S*_*f*_ increases, the maximum PoF $P_{f}^{\max}$ concomitantly increases, and the rate of this increase also rises. The results in Fig 4 indicate that the results of the proposed method are close to those of MCS and AK-MCS. As illustrated in Table 5, the maximum error between the proposed method and MCS is within 1%, which shows the good performance of the proposed method. By comparing the calculation results of CCM-MDRM and MCCM-MDRM, it can be found that the calculation results of MCCM-MDRM are the same as those of CCM-MDRM, while the number of iterations is significantly reduced, as shown in Table 6.

**Table 5:** Comparison of the maximum PoF $P_{f}^{\max}$ for different methods under different safety factors *S*_*f*_.

	*S*_*f*_ = 1.45	*S*_*f*_ = 1.48	*S*_*f*_ = 1.50	*S*_*f*_ = 1.52	*S*_*f*_ = 1.55
MCS	0.0200	0.0558	0.0990	0.1621	0.2943
AK-MCS	0.0202	0.0564	0.1010	0.1630	0.2934
*R*_*e*_	1.00%	1.08%	2.02%	0.56%	0.31%
CCM-MDRM	0.0199	0.0556	0.0987	0.1612	0.2923
*R*_*e*_	0.50%	0.36%	0.30%	0.56%	0.68%
MCCM-MDRM	0.0199	0.0556	0.0987	0.1612	0.2923
*R*_*e*_	0.50%	0.36%	0.30%	0.56%	0.68%

**Table 6:** Comparison of the iterations of different methods.

	λ=0.1	λ=0.3	λ=0.5	λ=0.7	λ=0.9
MCS	10^6^	10^6^	10^6^	10^6^	10^6^
AK-MCS (*n*_1_)	60	60	60	60	60
CCM-MDRM	101	34	19	14	9
MCCM-MDRM (*n*_2_)	79	28	17	12	7
$n_{2}/n_{1}$	131.67%	46.67%	28.33%	20%	11.67%

Table 6 shows the number of iterative calculations required by the proposed model for different control parameters *λ*. It can be seen in Table 6 that when the control parameter *λ* is small, the proposed model requires more iterations compared to AK-MCS. As the control parameter increases, the number of iterative calculations required by the proposed model drops rapidly, demonstrating that the efficiency of the proposed model is further enhanced. Moreover, the maximum PoF $P_{f}^{\max}$ for different control parameters *λ* under the safety factor *S*_*f*_ = 1.50 are shown in Table 7, showing good robustness of the proposed method.

**Table 7:** Comparison of the results of the proposed method for different control factors *λ.*

	λ=0.1	λ=0.3	λ=0.5	λ=0.7	λ=0.9
CCM-MDRM	0.0199	0.0199	0.0199	0.0199	0.0199
MCCM-MDRM	0.0199	0.0199	0.0199	0.0199	0.0199

### 4.3 Contact fatigue of the flexspline

In this example, the diameter of the flexspline indexing circle *d*_1_ is regarded as a random variable, the output torque *T* is regarded as an interval variable, and the rotational speed is set as *n*_*r*_ = 3000. The detailed distributions of the uncertain parameters are shown in Table 8.

**Table 8:** The distribution of the uncertain variables used in assessing the contact fatigue of the flexspline of the harmonic drive.

Variable	Distribution parameter 1	Distribution parameter 2	Variable type
*d*_1_ (mm)	50	0.5	Normal
*T* (N·m)	27	33	Interval

**Fig 5:**
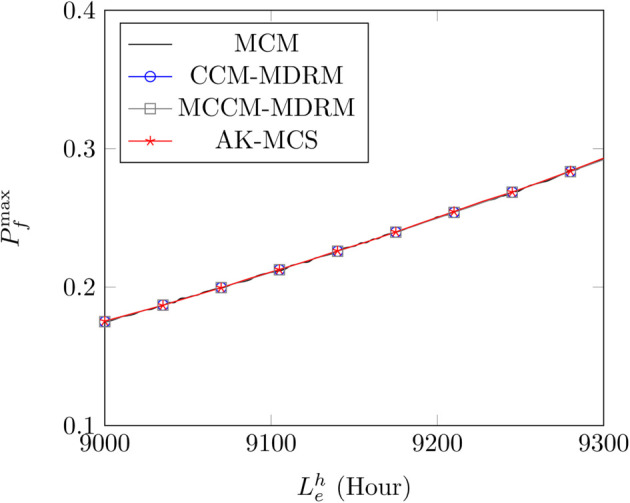
The variation of the maximum PoF $P_{f}^{\max}$ with the rated life $L_{h}^{e}$ for different methods.

Fig 5 and Table 9 show the results for different methods under the control parameter λ=0.1. As shown in Fig 5 and Table 9, the influence of the rated life $L_{h}^{e}$ is more nearly linear, which is due to the simple expression of the limit state function. By comparing the results of the proposed to those of MCS and AK-MCS, the good performance is demonstrated again as shown in Fig 5 and Table 9. Moreover, the same results are obtained in this example that with the increasing control parameters *λ*, less iteration times are needed, as shown in Tables 10 and 11.

**Table 9:** Comparison of the maximum PoF $P_{f}^{\max}$ for different methods under different rated life $L_{h}^{e}$.

	$L_{h}^{e}=9000$	$L_{h}^{e}=9100$	$L_{h}^{e}=9200$	$L_{h}^{e}=9300$
MCS	0.1748	0.2107	0.2500	0.2919
AK-MCS	0.1754	0.2109	0.2505	0.2927
*R*_*e*_	0.34%	0.09%	0.20%	0.27%
CCM-MDRM	0.1750	0.2106	0.2498	0.2921
*R*_*e*_	0.11%	0.05%	0.08%	0.07%
MCCM-MDRM	0.1750	0.2106	0.2498	0.2921
*R*_*e*_	0.11%	0.05%	0.08%	0.07%

**Table 10:** Comparison of the iterations of different methods.

	λ=0.1	λ=0.3	λ=0.5	λ=0.7	λ=0.9
MCS	10^6^	10^6^	10^6^	10^6^	10^6^
AK-MCS (*n*_1_)	36	36	36	36	36
CCM-MDRM	152	49	27	16	9
MCCM-MDRM (*n*_2_)	70	40	20	12	7
$n_{2}/n_{1}$	194%	90%	56%	33%	19%

**Table 11:** Comparison of the maximum PoF $P_{f}^{\max}$ for different control parameter *λ.*

	λ=0.1	λ=0.3	λ=0.5	λ=0.7	λ=0.9
CCM-MDRM	0.1748	0.1748	0.1748	0.1748	0.1748
MCCM-MDRM	0.1748	0.1748	0.1748	0.1748	0.1748

### 4.4 Tooth surface wear fatigue of the flexspline

In this example, only the output torque *T* is treated as an interval variable, and other parameters are regarded random variables, as detailed distributions are outlined in Table 12.

**Table 12:** The distribution of the uncertain variables used in assessing the tooth surface wear fatigue of the flexspline of the harmonic drive.

Variable	Distribution parameter 1	Distribution parameter 2	Variable type
*d*_1_ (mm)	20	2	Normal
*h*_*n*_ (mm)	0.36	0.0036	Normal
*b* (mm)	8	0.08	Normal
*K*	1.25	0.0125	Normal
*T* (N·m)	27	33	Interval

**Fig 6:**
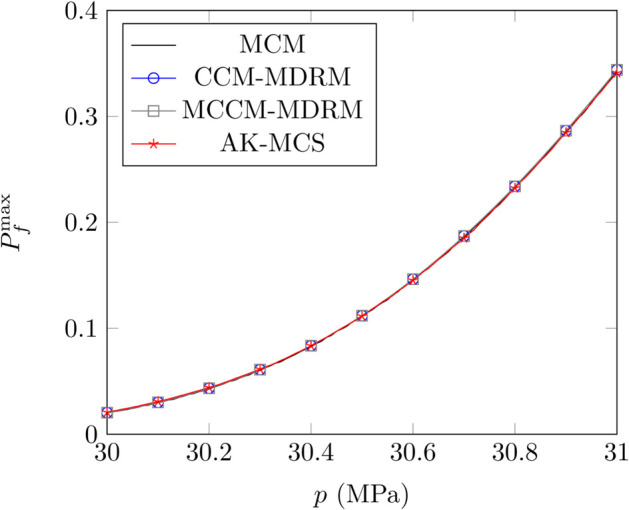
The variation of the maximum PoF $P_{f}^{\max}$ with the permissible specific pressure p for different methods.

The results of the tooth surface wear fatigue of the flexspline are shown in Fig 6 and Tables 13 and 15 when the control parameter is λ=0.1. As shown in Fig 6 and Table 13, the maximum error of the proposed method is 1%, while it is 2.54% for AK-MCS, which demonstrate the good robustness of the proposed method. Table 14 illustrates the iterations for different methods, and only 10% iterations of AK-MCS is required when the control parameter λ=0.9, which illustrates the efficiency of the proposed method. Moreover, the results shown in Table 15 indicate that the larger control parameter *λ* can improve the efficiency of the proposed model while maintaining the accuracy.

**Table 13:** Comparison of the maximum PoF $P_{f}^{\max}$ for different permissible specific pressure *p.*

	*p* = 30	*p* = 30.25	*p* = 30.50	*p* = 30.75	*p* = 31
MCS	0.0202	0.0511	0.1111	0.2089	0.3426
AK-MCS	0.0207	0.0524	0.1115	0.2077	0.3410
*R*_*e*_	2.48%	2.54%	0.36%	0.57%	0.47%
CCM-MDRM	0.0204	0.0516	0.1119	0.2098	0.3439
*R*_*e*_	1.00%	0.98%	0.72%	0.43%	0.38%
MCCM-MDRM	0.0204	0.0516	0.1119	0.2098	0.3439
*R*_*e*_	1.00%	0.98%	0.72%	0.43%	0.38%

**Table 14:** Comparison of the iterations of different methods.

	λ=0.1	λ=0.3	λ=0.5	λ=0.7	λ=0.9
MCS	10^6^	10^6^	10^6^	10^6^	10^6^
AK-MCS (*n*_1_)	40	40	40	40	40
CCM-MDRM	96	32	18	11	4
MCCM-MDRM (*n*_2_)	60	23	14	9	4
$n_{2}/n_{1}$	150%	58%	35%	23%	10%

**Table 15:** Comparison of the maximum PoF $P_{f}^{\max}$ for different control parameter *λ.*

	λ=0.1	λ=0.3	λ=0.5	λ=0.7	λ=0.9
CCM-MDRM	0.0204	0.0204	0.0204	0.0204	0.0204
MCCM-MDRM	0.0204	0.0204	0.0204	0.0204	0.0204

## 5 Conclusion

In this paper, the hybrid uncertainties of the harmonic drive is conducted. Dealing with hybrid uncertainty problem, the hybrid uncertainty analysis is decomposed into the iterative solution of probabilistic reliability and interval uncertainty problems, with the MCCM and MDRM methods employed to handle them respectively. By adopting the MCCM, issues of computational non-convergence (compared with HL-RF) and low computational efficiency (compared with CCM) can be effectively addressed. Moreover, the MDRM is used to convert the interval uncertainty problem into a function extremum solution problem, which greatly reduces the solution dimension of the problem. Due to the simplicity and comprehensibility of the theories underlying MCCM and MDRM, the proposed method only requires simple modifications to probabilistic reliability procedures to obtain hybrid uncertainty results of structures. By comparing the results of the proposed method with the AK-MCS and CCM-MDRM, it shows that the proposed method can achieve relatively accurate results with only 10% of the number of iterations required by AK-MCS. This method is simple and easy to implement, which greatly facilitates the engineering application of the proposed model.

In the reliability analysis of harmonic drives, the existence of interval variables alters the structural failure probability from a fixed value to a range. As cognition increases, interval variables transform into fuzzy or probabilistic variables; hence, distinct methods are required to assess the failure probability of harmonic drives. In this study, the probability-interval hybrid uncertainty of harmonic drives is explored, revealing divergent results across different failure modes. For example, in the flexspline’s tooth surface wear fatigue and fatigue fracture, a nonlinear relationship arises between failure probability and strength as strength decreases; by contrast, such nonlinearity is minimal in the flexspline’s contact fatigue. Furthermore, the proposed hybrid uncertainty method can be not only used in harmonic drives, but also other systems such as aerospace and power systems [43]. It enables precise and rapid quantification of reliability for complex systems in industrial or engineering scenarios, thereby providing critical quantitative tools for mechanical systems with intricate uncertainties.

Nevertheless, the present work has several limitations. Notably, the correlation between probabilistic and interval variables has not been considered in the analysis, and such correlations can also introduce deviations in calculation results. However, accounting for these correlations requires a thorough understanding of the harmonic drive. Additionally, correlations exist between the harmonic drive’s various failure modes, and such inter-mode correlations are equally significant [44]. Moreover, the failure and wear of the harmonic drive are influenced by time-varying external loads, thus making time-varying reliability a critical component of evaluation. Finally, at the analytical method level, the adoption of a reliability-based probabilistic analysis method may lead to result deviations for highly nonlinear problems. Furthermore, when handling multi-dimensional interval variable problems, the approximation accuracy of MDRM may be insufficient.

In summary, a fast hybrid uncertainty analysis method is employed to examine the hybrid uncertainty in harmonic drives. In future research, we will focus on the time-varying characteristics of harmonic drives, while also considering the impacts of correlations among internal parameters and between multiple failure modes.

## Supporting information

S1 File The original data of the curve graph. (CSV)
