# Correction: An efficient hybrid reliability analysis method with application to the harmonic drive

**DOI:** 10.1371/journal.pone.0337348

**Published:** 2025-11-19

**Authors:** Jingqi Cui, Di Zuo, Xiaoxi Men, Chongshuai Wang

The Funding statement for this article is incorrect. The correct Funding statement is as follows: This study was funded by the Aeronautical Science Foundation of China (ASFC; grant no. 202400020Y9001 to CW); the Dalian Science and Technology Innovation Fund (grant nos. 2023RQ063, 2024RQ050 to DZ); and the Department of Education of Liaoning Province (grant nos. JYTQN2023010, LJ2424101500006 to DZ; LJ212410150051 to XM).
